# The value of shock index in prediction of cardiogenic shock developed during primary percutaneous coronary intervention

**DOI:** 10.1186/s12872-018-0924-z

**Published:** 2018-10-01

**Authors:** Zhonghai Wei, Jian Bai, Qing Dai, Han Wu, Shuaihua Qiao, Biao Xu, Lian Wang

**Affiliations:** Department of Cardiology, Drum Tower Hospital, Medical School of Nanjing University, 321 Zhongshan Road, Nanjing, 210008 Jiangsu Province China

**Keywords:** Shock index, Myocardial infarction, Cardiogenic shock, Reperfusion

## Abstract

**Background:**

Shock index(SI) is a conventional predictive marker for haemodynamic state. Its breakpoint varies by different conditions according to previous studies. The current study was performed to evaluate the capability of SI in prediction of cardiogenic shock(CS) developed during primary percutaneous coronary intervention (pPCI).

**Methods:**

Total 870 patients of ST segment elevation myocardial infarction(STEMI) who were haemodynamic stable before pPCI were involved in the study. In this cohort, 625 consecutive patients composed analysis series and 245 consecutive patients composed validation series. Multivariate regression analysis was used to evaluate whether SI was a significant predictor of developed CS and Hosmer-Lemeshow test was used to assess the goodness of model fitness. Receiver-operating characteristics (ROC) analysis was used to compare the predictive capability of SI with other predictors. The sensitivity, specificity, accuracy, positive and negative predictive values of SI at different cutoff values was compared to identify a best breakpoint.

**Results:**

In the analysis series, SI and Killips classification were identified as independent predictors. ROC analysis demonstrated the diagnostic capability of SI was superior to pre-procedural systolic blood pressure(SBP) or heart rate(HR) alone (0.8113 vs 0.7582, *P* = 0.04 and 0.8113 vs 0.7111, *P* < 0.001). The diagnostic capability of SI was equivalent to that of combination of SBP, HR and Killips claasification(0.8133 vs 0.8137, *P* = 0.97). SI had a high specificity and low sensitivity. When the cutoff value was set at 0.93, the positive predictive value, negative predictive value and diagnostic accuracy was 42.6%, 95.1% and 87.4% respectively. In validation series, the area under ROC curve was 0.8245, which was similar to that in the analysis series. The positive predictive value, negative predictive value and diagnostic accuracy at the cutoff value of 0.93 was 53.8%, 93.2% and 88.9% respectively.

**Conclusions:**

SI has a high predictive accuracy for developing CS during pPCI in STEMI patients. It is an excellent exclusion diagnosis index rather than confirmative diagnosis index.

## Background

In the past three decades, the in-hospital and 1-year mortality of ST segment elevation myocardial infarction (STEMI) have been remarkably decreased due to timely revascularization [1]. However, the worsening of cardiac function after STEMI is still rising despite of optimal reperfusion and pharmacological therapy. The infarct-related heart failure will no doubt increase the long-term comorbidity and mortality, which may counterbalance the benefits from the timely reperfusion. Previous studies have revealed that approximate 50% of final infarct myocardium caused by reperfusion injury (RI) [2, 3]. RI is therefore regarded as the leading cause of infarct size extension after blood flow recovery of infarct-related artery (IRA), which could possibly lead to cardiogenic shock (CS) during primary percutaneous coronary intervention (pPCI). It has been reported that patients of STEMI complicated with CS have 30-day or in-hospital mortality as high as nearly 50% [4–7]. Hence, those stable patients of STEMI but probably developing to CS during pPCI should be identified in advance and it may provide the target patients to doctors to take measures for ease of RI.

Shock index (SI) is a marker assessing the haemodynamic state, which is calculated as heart rate (HR) divided by systolic blood pressure (SBP) [8]. Patients with elevated SI, even with normal blood pressure and heart rate, should be paid more attention for the high risk of shock. In the acute coronary syndrome (ACS) or STEMI patients cohort, SI has been proven the independent predictor of long-term major adverse cardiac events (MACE) or mortality [9–11]. Nevertheless, there are few studies on the efficacy of this marker in prediction of developing CS during emergency reperfusion. The current study was aimed to evaluate the predictive capability of CS developed during pPCI in the cohort of STEMI.

## Methods

### Study population

The study cohort was retrieved from the database of our center. The including criteria was as follows: (1) the patients were diagnosed STEMI; (2)there was no cardiogenic shock when admitted in emergency room; (3) the patients accepted PCI after emergency angiography. The exclusion criteria was as follows: (1)the patients presented cardiogenic shock when arrived emergency room;(2) the patients rejected emergency angiography; (3) the patients did not need emergency revascularization or need emergency coronary artery bypass graft (CABG) surgery; (4)the patients were deployed prophylactic IABP before revascularization.

From January 2010 to May 2017, total 1250 STEMI patients were admitted in our hospital. 250 patients were excluded because they did not accepted emergency PCI due to over the time window of emergency revascularization. 59 patients were excluded due to cardiogenic shock when admitted in emergency room. 37 patients were excluded for sake of prophylactic use of IABP. 23 patients were excluded because of referral to emergency CABG or referral to elected procedure. 11 patients were excluded due to refusal of emergency angiography. Therefore, the remaining 870 patients were eligible for the study cohort. The study population consisted of 2 series: 1 analysis series (625 consecutive patients for analysis and identification of predictive capability) and 1 validation series (245 consecutive patients for validation the predictive capability).

### Procedure details

All the patients with acute chest pain in emergency room accepted ECG within 10 min. STEMI was defined as new onset of ST segment elevation at the J point in at least 2 contiguous leads of more than 2 mm in men or more than 1.5 mm in women in V2 and V3 lead and/or of more than 1 mm in other leads. The presentation of new left bundle branch block was considered equivalent to STEMI [12]. Cardiogenic shock was defined that the systolic blood pressure of the patients is below 90 mmHg more than 30 min or the inotropic agents are needed to maintain the systolic blood pressure above 90 mmHg accompanied with pulmonary congestion and/or peripheral perfusion impairment [13].

The patients ready to accept primary PCI were administered a loading dose of aspirin 300 mg and ticagrelor 180 mg before the procedure. Clopidogrel 600 mg was given if ticagrelor was contraindicated or unavailable. After a radial or femoral artery puncture, a 6F sheath was inserted. Heparin was administered at a dose of 70–100 IU/kg, while tirofiban, urokinase or argatroban were used if necessary. Thrombus aspiration catheter was used if it was considered high burden of thrombus under angiography. After blood flow recovery of IRA, the stent was deployed immediately or delayed according to the discretion of coronary lesions and interventionists’ experience. If CS occurred after the reperfusion of IRA, rescue IABP support was transfemorally placed preferentially. If the patients were not suitable for IABP, inotropic agents were alternative. All of the procedures were accomplished by experienced and qualified interventionists.

### Statistics

Continuous normally distributed variables were shown as mean ± standard deviation (mean ± SD) and were compared using T-test between two groups. While those that were not normally distributed were presented as median (M) and interquartile range (IQR) and compared using Wilcoxon rank-sum test between two groups. Categorical variables were shown as frequencies and percentages and were compared with χ^2^ test or Fisher exact test. In the regression analysis, the following variables in the analysis series were set in the univariate regression analysis initially: age, gender, pre-procedural systolic blood pressure(SBP), pre-procedural heart rate(HR), Killips classification, total ischemic duration, multiple vessel disease, extensive anterior myocardial infarction(MI), infarct related artery(IRA), hypertension, diabetes, dyslipidemia, prior MI, prior stroke, smoking hobby, serum creatinine. The variables significant in univariate analysis were subsequently set in the multivariate analysis. The variables were selected using backwards method. The regression models were calibrated with Hosmer-Lemeshow χ^2^ test for the goodness of fit. Thereafter, the significant covariates were tested for the accuracy with receiver-operating characteristics (ROC) analysis. The area under curve (AUC) was calculated to compare the diagnostic capability of the predictors. Specificity, sensitivity and Youden index (specificity+sensitivity-1) were calculated for identification of a reasonable cutoff value. In the validation series, the predictors were analyzed with ROC in order to identify the predictive value. The statistical analysis was performed by Stata version 12.0 (StataCop., College Station, Texus, USA). All the tests were 2 sided. Values of *P* < 0.05 were considered statistically significant.

## Results

### Characteristics of study population

Total 870 patients were valid for the current study. The median age was 65y with interquartile range of 55y-74y. There were 686 male patients (78.9%) and 184 female patients(21.1%). 769 patients were hemodynamic stable during pPCI(Non Shock Group), whereas 101 patients had developed CS during the procedure(Developed Shock Group). Compared with Developed Shock Group, Non Shock Group had lower proportion patients with prior stroke (*P* < 0.001), higher EF value (*P* = 0.03), higher pre-procedural SBP(*P* < 0.001) and lower pre-procedural HR(*P* = 0.008). Furthermore, more patients had left circumflex branch(LCX) or obtuse marginal branch(OM) as IRA and less patients had right coronary artery(RCA) as IRA in the Non Shock Group(P < 0.001). As regard to pharmacological therapy, there were more patients taking β-blocker in Non Shock Group in comparison with that in Developed Shock Group(P < 0.001), while other medication was no different between two groups (Table 1).

**Table 1:** Characteristic of patients cohort

	Non-Shock (*n* = 769)	Developed Shock (*n* = 101)	*P* value
Age,year[M(IQR)]	64(55–74)	63(55–73)	0.52
Male sex,n(%)	610(79.3)	76(75.6)	0.35
Anterior myocardial infarction,n(%)	389(50.6)	58(57.6)	0.2
Hypertension,n(%)	505(65.7)	69(68.8)	0.60
Diabetes,n(%)	215(28.0)	34(33.3)	0.23
Prior Stroke,n(%)	101(13.1)	30(30.3)	< 0.001
Hyperlipidemia,n(%)	64(9.7)	9(9.1)	0.84
Smoke,n(%)	450(58.5)	53(52.1)	0.25
Prior myocardial infarction, n(%)	73(9.53)	8(7.98)	0.61
Creatinine,μmol/L[M(IQR)]	72(62–87)	69(60–81)	0.22
EF,%[M(IQR)]	47(41–50)	44(40–48)	0.03
Triglyceride, mmol/L[M(IQR)]	1.38(1.00–2.05)	1.39(0.90–2.10)	0.72
Cholesterol, mmol/L[M(IQR)]	4.28(3.63–4.98)	4.49(3.83–4.88)	0.46
LDL-C, mmol/L[M(IQR)]	2.31(1.84–2.80)	2.29(1.91–2.79)	0.93
HDL-C, mmol/L[M(IQR)]	0.93(0.76–1.14)	0.91(0.77–1.11)	0.79
Pre-procedure SBP, mmHg[M(IQR)]	123(112–138)	104(96–108)	< 0.001
Pre-procedure HR, bpm[M(IQR)]	79(69–89)	91(82–100)	0.008
Total ischemic time,min[M(IQR)]	342(234–610)	360(267–713)	0.26
Killips class II/III, n(%)	185(24.0)	30(28.7)	0.50
Double vessel disease, n(%)	310(40.3)	34(33.3)	0.20
Triple vessel disease, n(%)	235(30.6)	40(39.4)	0.07
IRA			
LAD, n(%)	389(50.6)	58(57.6)	0.20
LCX/OM, n(%)	123(16)	1(0.99)	< 0.001
RCA, n(%)	195(25.4)	43(42.4)	< 0.001
PDA/PL, n(%)	65(8.5)	0	–
PTCA, n(%)	12(1.51)	0	–
Stents			
Sirolimus, n(%)	481(62.6)	58(57.6)	0.32
Everolimus, n(%)	231(30.1)	30(30.3)	0.95
Zotarolimus, n(%)	155(20.1)	24(24.2)	0.40
Paclitaxel, n(%)	6(0.76)	0	–
Mediction			
Aspirin, n(%)	769(100)	101(100%)	1.00
Clopidogrel, n(%)	516(67.1)	64(63.4)	0.45
Ticagrelor, n(%)	253(32.9)	37(36.6)	0.45
ACEI/ARB, n(%)	511(66.5)	64(63.6)	0.54
β-blocker, n(%)	606(78.8)	70(69.4)	< 0.001
Spironolactone, n(%)	442(57.5)	63(62.7)	0.35
Diuretics, n(%)	359(46.7)	55(54.5)	0.14
Statin, n(%)	767(99.8)	100(99.6)	0.31

*EF* ejection fraction, *LDL-C* low density lipoprotein cholesterol, *HDL-C* high density lipoprotein cholesterol, *SBP* systolic blood pressure, *HR* heart rate, *IRA* infarct related artery, *LAD* left anterior descending branch, *LCX* left circumflex branch, *OM* obtuse marginal branch, *RCA* right coronary artery, *PDA* posterior descending artery, *PL* posterior branch of left venticule, *ACEI* angiotensin converting enzyme inhibitor, *ARB* angiotensin receptor blocker

### Identification of relevant risk factors

In the analysis series, 72 patients were subjected to the CS during primary PCI. We took CS as dependent variable, the following factors as independent variables: age, sex category, pre-procedural SBP and pre-procedural HR, Killips classification, total ischemic duration, multiple vessel disease, extensive anterior MI, IRA, renal function, prior related history including hypertension, diabetes, dyslipidemia, MI, stroke and smoking habit. On univariate analysis, pre-procedural SBP and pre-procedural HR, Killips classification were the significant variables. Prior history of hypertension had a trend toward to statistical significance (Table 2).

**Table 2:** Univariate regression analysis for the risk factors

Variables	OR	SE	*P* value	95% CI
Age	1.00	0.01	0.57	[0.99 1.03]
Female sex	1.14	0.34	0.65	[0.64 2.04]
Pre-Procedural SBP	0.94	0.01	< 0.01	[0.92 0.96]
Pre-Procedural HR	1.05	0.01	< 0.01	[1.03 1.06]
Total ischemic duration (per 1 h change)	1.01	0.01	0.51	[0.98 1.02]
Killips classification				
Killips = 2 vs =1	2.45	0.67	0.001	[1.43 4.19]
Killips = 3 vs =1	6.20	3.44	0.001	[2.09 18.4]
Multiple vessel disease	1.44	0.39	0.18	[0.85 2.43]
Extensive anterior MI	0.89	0.26	0.69	[0.50 1.58]
Prior MI	0.96	0.73	0.95	[0.22 4.25]
Prior hypertension	0.61	0.15	0.05	[0.37 1.00]
Prior diabetes	0.72	0.22	0.28	[0.40 1.30]
Prior stroke	1.64	0.53	0.13	[0.87 3.10]
Prior dyslipidemia	1.06	0.45	0.89	[0.46 2.44]
Smoking	0.96	0.24	0.88	[0.59 1.58]
Serum creatinine	1.00	0.01	0.58	[0.99 1.01]

*SBP* systolic blood pressure, *HR* heart rate, *MI* myocardial infarction

In multivariate analysis, the above statistical significant variables together with some clinical significant variables including age, total ischemic duration, multiple vessel disease were set in the multivariate regression analysis. Table 3 showed the pre-procedural SBP, pre-procedural HR and Killips classification were the independent predictors. Hosmer-Lemeshow test demonstrated the model was well fitted (χ^2^ = 6.43, *P* = 0.599).

**Table 3:** Multivariate regression analysis for risk factors

Variables	OR	SE	*P* value	95% CI
Pre-Procedural SBP	0.95	0.01	< 0.01	[0.93 0.96]
Pre-Procedural HR	1.05	0.01	< 0.01	[1.03 1.07]
Killips classification				
Killips = 2 vs =1	2.34	0.76	0.01	[1.24 4.44]
Killips = 3 vs =1	5.30	2.86	0.02	[1.27 22.1]

*SBP* systolic blood pressure, *HR* heart rate

### Model fit of SI

**Fig. 1:**
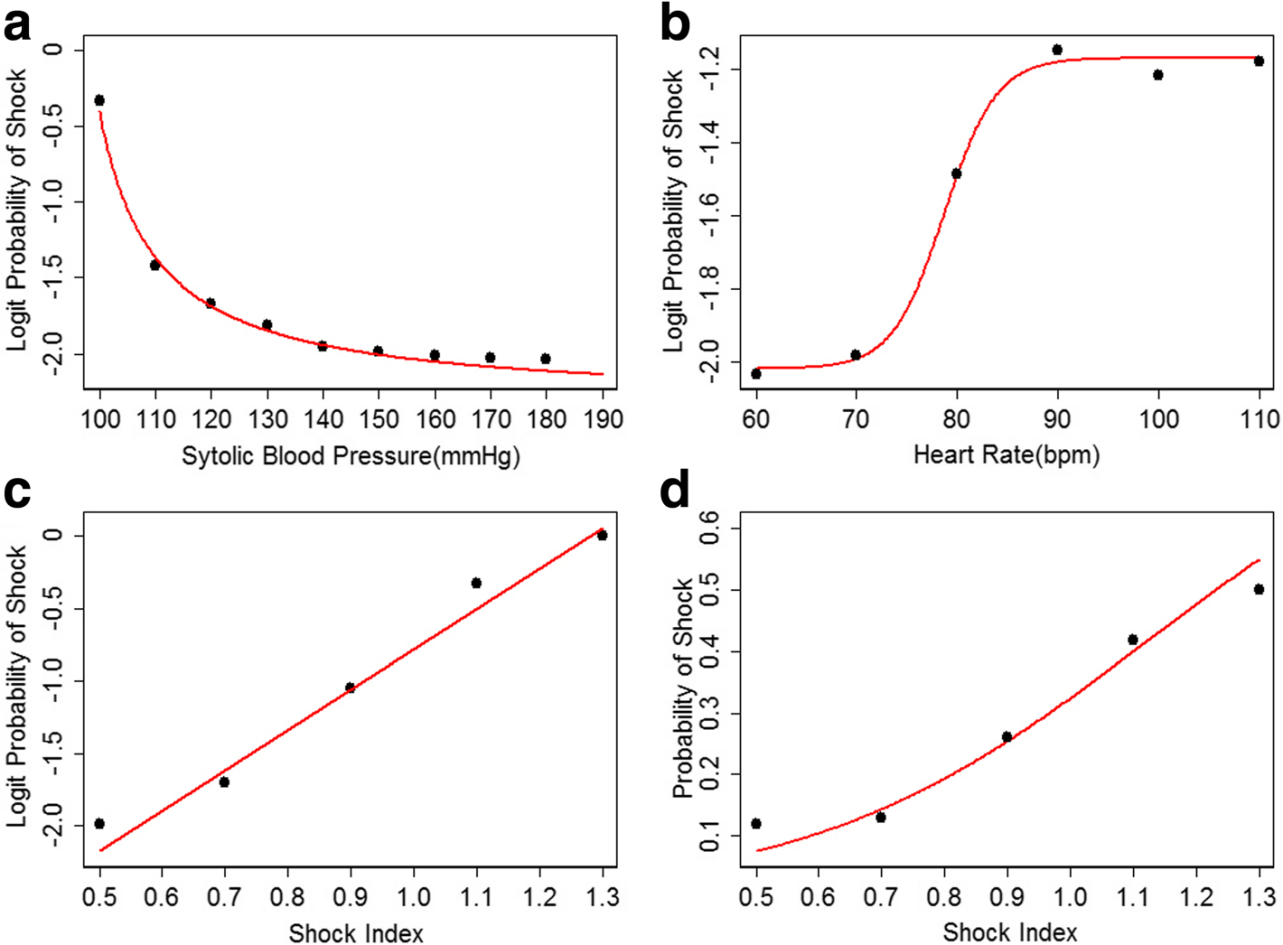
(**a**) The logit probability of developing CS showed inverse proportional to the pre-procedural SBP. (**b**) The relationship between pre-procedural HR and logit probability of developing CS formed a sigmoid curve. (**c**) The logit probability of developing CS was positive linear correlated to the SI. (**d**) The relationship between probability of developing CS and SI was fitted into a logistic function. The curve shown in the graph was just at the rapid descending part of the logistic curve, which was very close to a line. SBP: systolic blood pressure; HR: heart rate; SI: shock index; CS: cardiogenic shock

The regression model revealed the risk of developing CS was positively correlated with pre-procedural HR and negatively correlated with pre-procedural SBP. Furthermore, the scatter plot showed the relationship between logit probability of shock and pre-procedural SBP was nonlinear and a inverse proportional function was well fitted (*P* < 0.001,adjust R square = 0.9904)(Fig. 1a). The relationship between logit probability of shock and pre-procedural HR was also nonlinear and a logistic function was fitted (*P* < 0.001, adjust R square = 0.9973)(Fig. 1b). SI was formulated as the ratio of pre-procedural HR to pre-procedural SBP. The logit probability of shock was positively linear correlated with SI (P < 0.001, adjust R square = 0.9549). The relationship between probability of shock and SI was located at the rapid rise part of a sigmoid curve and the logistic function was well fitted (P < 0.001, adjust R square = 0.9898) (Fig. 1c and d). Regression analysis demonstrated that SI was a significant independent predictor (Hosmer-Lemeshow χ^2^ = 8.12, *P* = 0.42)(Table 4). However, there was no interplay between SI and Killips classification no matter in CS cohort, non-CS cohort or global cohort. That meant Killips classification was not able to further promote the discriminability of SI.

**Table 4:** Multivariate regression analysis for shock index

Variables	OR	SE	*P* value	95% CI
Shock index (per 0.1 change)	1.93	0.16	< 0.01	[1.64 2.28]
Killips classification				
Killips = 2 vs =1	2.21	0.69	0.01	[1.21 4.06]
Killips = 3 vs =1	5.94	3.92	0.01	[1.63 21.7]
Shock index * Killips			> 0.05^#^	

^#^ *P* > 0.05 indicated there was no significant interaction between shock index and Killips classification in the multivariate regression analysis

### Diagnostic capability assessment

**Fig. 2:**
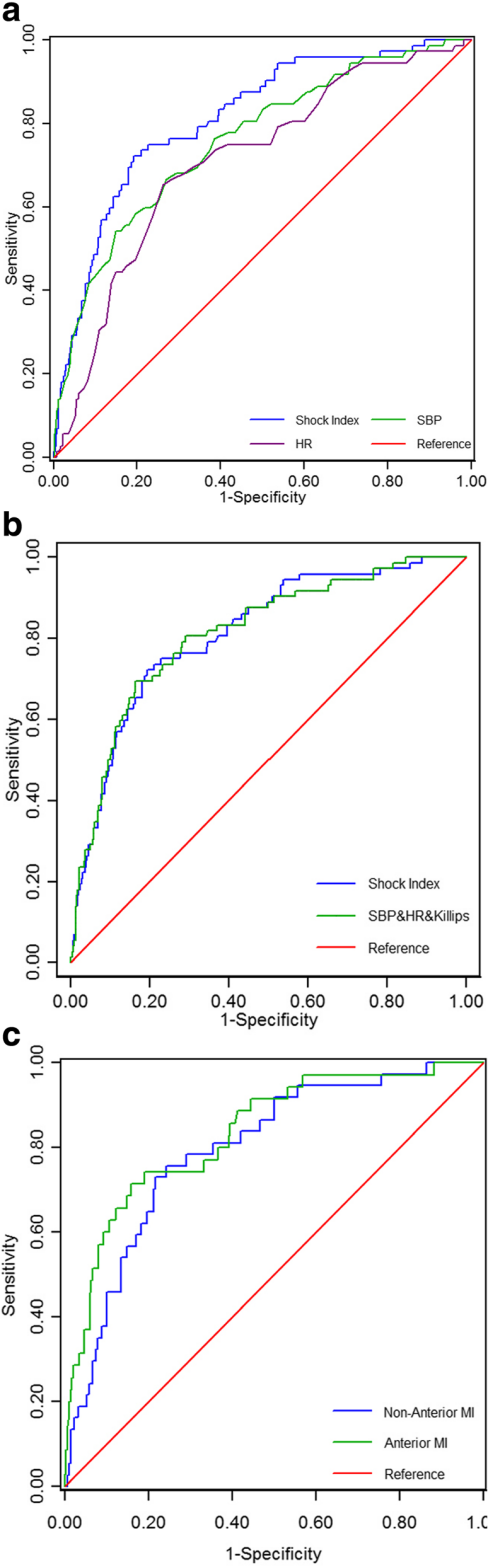
(**a**) SI had a better diagnostic capability than pre-procedural SBP or pre-procedural HR alone. (**b**) The diagnostic capability of SI was similar to that of the combination of pre-procedural SBP and pre-procedural HR and Killips classification. (**c**) The diagnostic capability of SI had no difference between non-anterior MI subgroup and anterior MI subgroup. SI: shock index; SBP: systolic blood pressure; HR: heart rate; MI: myocardial infarction

With the calibration of ROC analysis, the AUC of SI was significantly higher than that of pre-procedural SBP(0.8113 vs 0.7582, *P* = 0.04) and pre-procedural HR(0.8113 vs 0.7111, *P* < 0.001) respectively(Fig. 2a). The AUC of SI was similar to the AUC of combination of SBP, HR and Killips classification(0.8133 vs 0.8137, *P* = 0.97) (Fig. 2b). Moreover, the AUC of SI in anterior MI patients was not significantly different from that in non-anterior MI patients(0.8332 vs 0.7944, *P* = 0.46)(Fig. 2c).

The diagnostic parameters were calculated at different cutoff of SI (Table 5). Of note, SI had a high specificity and low sensitivity because of the relatively low incidence of developing CS during pPCI. In other words, it was suitable for exclusion diagnosis. When the cutoff was between 0.90~ 0.95, the accuracy were all above 85% and the negative predictive value were higher than 90%. If the cutoff was set at 0.93, the negative predictive value was as high as 95%.

**Table 5:** The diagnostic capability assessment of shock index

Cutoff	Sensitivity(%)	Specificity(%)	Youden index(%)	Accuracy(%)	Positive predictive value(%)	Negative predictive value(%)
0.90	31.9	93.7	25.6	86.6	39.7	91.4
0.91	30.6	94.0	24.6	86.7	40.0	91.2
0.92	29.2	94.9	24.1	87.4	42.9	91.2
0.93	27.8	95.1	22.9	87.4	42.6	95.1
0.94	26.4	95.3	21.7	87.4	42.2	90.9
0.95	22.2	96.2	18.4	87.7	43.2	90.5

### Predictive capability validation of SI

**Fig. 3:**
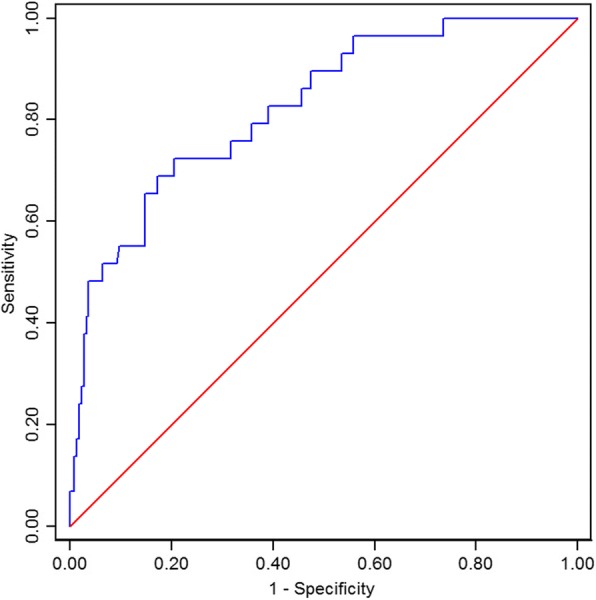
The ROC curve of shock index in the validation series

**Fig. 4:**
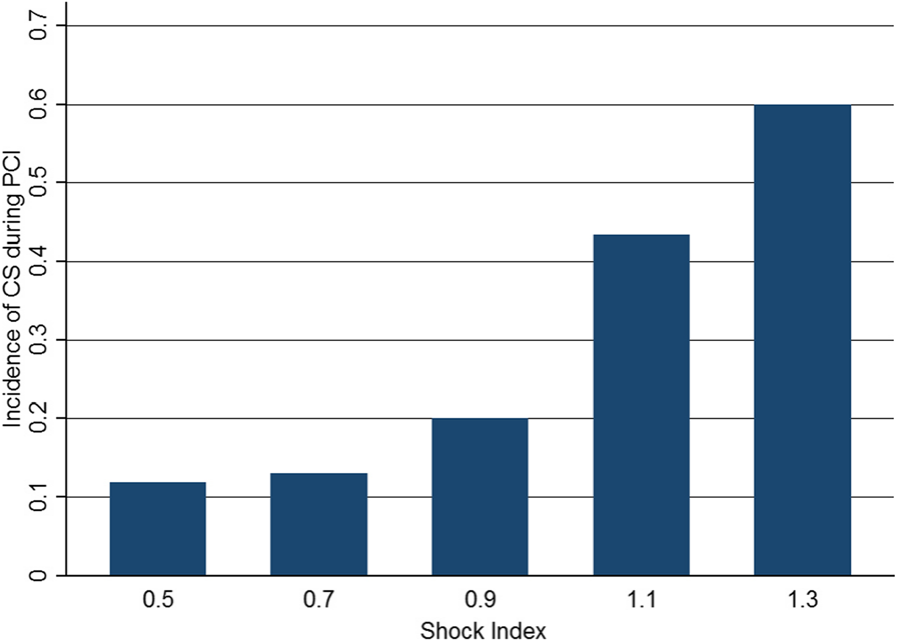
The probability of CS developed during pPCI at different cutoff values of SI in the validation series. CS: cardiogenic shock; SI: shock index: pPCI: primary percutaneous coronary intervention

In the validation series, total 29 patients had undergone CS during primary PCI. Logistic regression analysis demonstrated that the odd ratio of SI (per 0.1 changes) for predicting this event was 1.94(95% CI: 1.54–2.46). Hosmer-Lemeshow test revealed a excellent model fit(χ^2^ = 8.57, *P* = 0.38). The ROC curve of SI was depicted in Fig. 3. The AUC of this curve was 0.8245(95% CI: 0.7441–0.9048). The incidence of developing CS at different breakpoints was shown in Fig. 4. If the cutoff value was set at 0.93, the sensitivity, specificity, positive predictive value and negative predictive value was 48.3%, 94.4%, 53.8% and 93.2% respectively. The accuracy and Youden index was 88.9% and 42.7% respectively.

## Discussion

SI is a reliable predictor for early shock in different situation, such as trauma, infection, pulmonary embolism, which is usually set 0.9 as the threshold of elevation [14–18]. However, there are several other cutoff values in different studies [17, 19, 20], which means the diagnostic capability of SI varies over different conditions. Theoretically, SI should be more sensitive in reflexing the pre-shock state because heart rate usually elevates before the systolic blood pressure goes down as a compensatory response. Surprisingly, the consequence in our data was beyond our expectation.

Pre-procedural SBP, pre-procedural HR and Killips classification have been identified independent predictors of developing CS during emergency reperfusion in the current study, which is consistent with the previous findings [21, 22]. Further analysis showed the relationship of logit probability of CS with pre-procedural SBP and pre-procedural HR were both not linear. The scatter dots were fitted into an inverse proportional function in the former relationship and a logistic function in the latter relationship. Despite the monotone change could make SBP and HR predictors, there were flat parts in the both curves, which probably caused the makers less sensitive. On the contrary, SI had much better feature in this regard. The logit probability of shock was positive linear related to SI, which was an ideal relationship for the binary variables model. A recent study performed by Laust Obling et al. demonstrated that the odd ratio was 1.26 for per 10% change in the patients developing CS after leaving catheter laboratory [22]. In our data, the odd ratio was 1.93 for per 10% change. Of note, SI was not an independent predictor in Laust Obling’s study, while it did in our study. The leading cause may be the ejection fraction (EF) was set in the regression analysis in the previous study. EF was highly associated with CS and probably masked the effect of SI. But EF was not available before pPCI in our setting.

ROC analysis proved that SI had a better diagnostic capability than either SBP or HR alone, while it had an equivalent diagnostic capability with the combination of SBP, HR and Killips classification. Killips classification could not further improve the diagnostic capability due to no interaction between SI and Killips classification. Therefore, we had reason using SI instead of the other independent predictors. The AUC of SI in the analysis series was highly close to that in the validation series, which implied SI was a reliable predictor. Nonetheless, SI had a high specificity and low sensitivity despite the predictive accuracy was above 85%. The sensitivity and positive predictive value varied markedly, while the specificity and negative predictive value remained as high as about 95%. The incidence of CS developed during pPCI in the study cohort influenced the sensitivity and positive predictive value. Generally speaking, the incidence of developing CS was not very high however. It meant SI was not a good index for confirmative diagnosis but a good index for exclusion of developing CS.

In the previous studies, the cutoff values were arbitrary and also varied over different settings. Nevertheless, SI norms change by age and gender just because the blood pressure and heart rate varies by age and gender [18, 23]. SI declines about 0.01~ 0.02 per every 5 years in male population and about 0.02~ 0.03 per every 5 years in female population [18], which forms a slow declined curve. In other words, the same threshold might not be sensitive as to the aged population. Several studies have used age modified SI (age×SI) as a better predictor to offset the disadvantage of SI [24–26]. In our data, the breakpoint of SI was set at 0.93 as an ideal threshold for exclusion of developing CS. We did not use age modified SI just because age was not significant predictors. Moreover, we also attempted to modified SI using Killips classification to create a novel and better index, but failed in the end, which was mainly due to no interaction between SI and Killips classification.

## Conclusions

According to the current study, SI had advantage in prediction of developing CS during pPCI for STEMI patients. It had an excellent negative predictive capability, but the positive predictive capability was not as so good.

### Limitations

Firstly, the negative predictive value of SI had been proven approximate 95% by analysis and validation series. However, the difference of positive predictive value between analysis series and validation series was markedly due to a bit small sample size of validation series. Secondly, left main artery(LM) as IRA is a strong predictor of developing CS, which has been reported in previous study [22]. In our center, all the patients with LM as IRA had accepted prophylactic IABP support, which exclude these patients from current study. Therefore, we could not investigate the possible interaction of LM with SI. Thirdly, SI norms changes by age as aforementioned. We did not analyze the predictive value by age stratification due to age not being significant covariate by regression analysis. Maybe the age range in the data was not large enough or the analysis series sample was not large enough for SI to discriminate the diagnostic capability in different age groups. The last but not least, diabetes usually worsens the prognosis of the patients with acute myocardial infarction accompanied with multivessel disease [27, 28]. It may affect the anti-apoptotic properties of atherosclerotic plaques and reduce the mobilization of stem cells to repair the damaged myocardial tissue [29, 30]. However, in the current study, diabetes did not play an important role in developing CS during primary PCI. It may be considered that diabetes influences the long-term prognosis rather than instant consequence of acute myocardial infarction. Moreover, incretin, a novel antidiabetic drug, has been identified a protective effect on the cardiovascular events. It could improve the cardiovascular prognosis of diabetic patients by pleiotropic effect [27, 28]. In our patient cohort, we did not have the detail percentage of the patients who had accepted incretin therapy, which preclude us to evaluate whether incretin therapy could protect the patients against developing CS during primary PCI.

## Abbreviations

ACEI: Angiotensin converting enzyme inhibitor
ACS: Acute coronary syndrome
ARB: Angiotensin receptor blocker
AUC: Area under curve
CABG: Coronary artery bypass graft
CS: Cardiogenic shock
HR: Heart rate
IABP: Intra-aortic balloon counterpulsation
IRA: Infarct-related artery
LAD: Left anterior descending branch
LCX: Left circumflex branchor
MACE: Major adverse cardiac events
MI: Myocardial infarction
OM: Obtuse marginal branchas
PDA: Posterior descending artery
PL: Posterior branch of left venticule
pPCI: Primary percutaneous coronary intervention
RCA: Right coronary artery
ROC: Receiver-operating characteristics
SBP: Systolic blood pressure
SI: shock index
STEMI: ST segment elevation myocardial infarction
